# Curcumin Changed the Number, Particle Size, and miRNA Profile of Serum Exosomes in Roman Laying Hens under Heat Stress

**DOI:** 10.3390/genes15020217

**Published:** 2024-02-08

**Authors:** Kai Kang, Wen Gao, Yanfeng Cui, Mei Xiao, Lilong An, Jiang Wu

**Affiliations:** College of Coastal Agricultural Sciences, Guangdong Ocean University, Zhanjiang 524088, China; kangkai610@126.com (K.K.);

**Keywords:** serum exosomes, miRNAs, laying hens, heat stress, curcumin

## Abstract

Exosomes have the ability to transport RNA/miRNAs and possess immune modulatory functions. Heat stress, a significant limiting factor in the poultry industry, can induce oxidative stress and suppress the immune responses of laying hens. In this study, we investigated the expression profiles of serum exosomes and their miRNAs in Roman laying hens who were fed a diet with either 0 or 200 mg/kg curcumin under heat stress conditions. The numbers of exosomes were significantly higher in both the HC (heat stress) and HT (heat stress with 200 mg/kg curcumin) groups compared to the NC (control) group and NT (control with 200 mg/kg curcumin) group (*p* < 0.05). Additionally, we observed that the most prevalent particle diameters were 68.75 nm, 68.25 nm, 54.25 nm, and 60.25 nm in the NC, NT, HC, and HT groups, respectively. From our sRNA library analysis, we identified a total of 863 unique miRNAs; among them, we screened out for subsequent bioinformatics analysis a total of 328 gga-miRNAs(chicken miRNA from the miRbase database). The KEGG pathways that are associated with target genes which are regulated by differentially expressed miRNAs across all four groups at a *p*-value < 0.01 included oxidative phosphorylation, protein export, cysteine and methionine metabolism, fatty acid degradation, ubiquitin-mediated proteolysis, and cardiac muscle contraction. The above findings suggest that curcumin could mitigate heat-induced effects on laying hens by altering the miRNA expression profiles of serum exosomes along with related regulatory pathways.

## 1. Introduction

Exosomes, a subtype of extracellular vesicles, are released under both pathological and physiological conditions [1]. They facilitate the transportation of lipids, RNA/miRNAs, and proteins, while also possessing immunomodulatory functions [2]. Moreover, they impede chromatin interaction in target cells and transmit inflammatory, apoptotic, or regenerative signals through RNAs [3]. The high concentration and stability of miRNAs in exosomes make them ideal biomarkers compared to free miRNAs when analyzing bodily fluids [4]. Serum exosomal miRNAs have demonstrated significant potential as diagnostic biomarkers for individuals with abnormal physical conditions [5], with specific serum exosomal miRNAs already having been identified as novel biomarkers for clinical diagnosis. For instance, miR-34a has been proposed as a potential biomarker for enhancing the diagnostic efficiency of ovarian cancer (OC) [6], while miR-22-3p and miR-320a have been associated with patients suffering from endometriosis [7]. Exosomes frequently shuttle between different tissues and cells, providing a bridge for the communication between tissues and cells [8]. In particular, miRNAs that are carried in exosomes can not only be used as markers of tissues and cells in different physiological states but also regulate the gene expression of target cells and the life activities of related tissues and organs [9,10]. This means that exosomes and their specific miRNAs can play an important role in the physiological and pathological activities of the body, such as cell development and proliferation, tissue damage repair, and organ life activity regulation. 

In recent years, the poultry industry has encountered a significant challenge, attributed to global warming resulting in heat stress. This stress induces oxidative stress, diminishes poultry’s antioxidant defenses, and leads to a noticeable decline in the feed intake and egg quality among hens. Our previous study demonstrated that dietary supplementation of curcumin to laying hens under heat stress can enhance their antioxidant status and alleviate adverse environmental conditions [11]. Curcumin possesses the ability to improve production quality by boosting immunity and antioxidation capacity, making it an economical feed ingredient with no residues or side effects [12,13,14,15]. Studies have confirmed that curcumin could reduce the oxidative stress index in tissues and organs [16], had a positive effect on the antioxidant capacity and pathological change score of the body [17], and could regulate the miRNA expression profiles of tissues and cells by controlling target genes and their corresponding biological functions [18]. The endogenous miRNA-mediated gene silencing mechanism inhibits cell protein denaturation, where heat stress plays a positive inhibitory role [19]. Therefore, we hypothesize that certain serum exosomal miRNAs may exhibit distinct expression patterns that partially elucidate the positive effects of curcumin on Roman laying hens who are fed a diet supplemented with curcumin and who are under heat stress.

The present study aims to investigate the impact of heat stress on serum exosomes in laying hens through controlled artificial environmental simulation. Second-generation sequencing technology will be utilized to explore the influence of heat stress and curcumin on miRNA profiling of serum exosomes, with the objective of elucidating the mechanisms underlying oxidative stress that is induced by heat stress and examining how curcumin mitigates oxidative damage in laying hens under such conditions.

## 2. Materials and Methods

### 2.1. Experimental Chickens and Curcumin

The Roman chicks were purchased from Guangdong XiYing Poultry Co., Ltd. (Guangzhou, China), and fed in the animal room of the animal hospital of Guangdong Ocean University following the Lohmann Tierzucht GmbH Manual (https://www.ltz.de/en/downloads/management-guides.php (accessed on 27 May 2023)). The curcumin was purchased from Xi’an JinLv Biological Engineering Technology Co., Ltd. (Xi’an, China), purity > 97% (Lot Number: BW2017010A).

Twenty-four 26-week-old laying hens with similar weight were selected and randomly divided into four groups (six hens in one group): Control group (NC), Control with curcumin added group (NT), Heat stress group (HC), and Heat stress with curcumin added group (HT). The temperature of NC and NT groups was controlled at 25 ± 1 °C, and humidity was 65–75%. Temperature of HC and HT groups was controlled at 32 ± 1 °C during 10:00–16:00 and 25 ± 1 °C in the other hours every day, and humidity was 65–75%. The NC group and HC group were fed with general basic diet following the Lohmann Tierzucht GmbH Manual, while the NT and HT group were supplemented with 200 mg/kg (curcumin/diet) of curcumin in addition to basic diet. The chickens were fed regularly with their assigned diet, free availability of tap water, and excreta was cleaned at 18:00 every day. The light duration was 16 h/d, 10–15 lx. 

### 2.2. Blood Collection and Exosome Isolation

After a period of 30 days, blood samples were collected from all hens via wing vein puncture, following the approved protocols by The Animal Care and Use Committee at Guangdong Ocean University (Zhanjiang, China). The blood samples of each group (consisting of 6 hens) were collected in evacuated blood tubes and left at room temperature for 1 h. Subsequently, they were centrifuged within 10 min at 2000× *g* and 4 °C to obtain serum. Plasma exosomes were isolated and characterized as described below: Initially, the serum was transferred to a new tube and centrifuged at 2000× *g* for 10 min at 4 °C. Then, it was carefully transferred again to another tube and subjected to centrifugation at 10,000× *g* for 30 min at the same temperature. The resulting supernatant was diluted with PBS in a ratio of 1:1 and filtered using a Millipore filter (R6BA09493) with a pore size of 0.45 m to collect the filtrate. This filtrate was then transferred into another centrifugal tube and subjected to further centrifugation at −80 °C for an additional duration of 70 min under conditions of 110,000× *g* using Beckman Optima L-100XP equipment. The pellet obtained after this process was suspended in pre-cooled PBS (1 × PBS) with a volume of 10 mL before being subjected once more to centrifugation under similar conditions to earlier (−80 °C; 110,000× *g*; 70 min). Finally, the enriched population containing exosomes that was present in the pellet was resuspended in 50 mL PBS (Sangon Biotech (Shanghai, China), E607008) and stored at −80 °C.

### 2.3. Transmission Electron Microscopy

The exosomes obtained through ultra-fast centrifugation were extracted from a 5 uL sample and diluted to 10 uL prior to detection. Subsequently, a volume of 10 uL of the sample was applied onto a copper grid for a duration of 1 min, followed by absorption of the excess liquid using filter paper. Then, 10 uL of urani-um dioxygen acetate (phosphotungstic acid) was added to the copper grid for precipitation over a period of 1 min, with subsequent absorption using filter paper. Finally, the samples were air-dried at room temperature for several minutes before examination under an electron microscope operating at an acceleration voltage of 80 kv on FEI Tecnai Spirit TEM T12.

### 2.4. Nanoparticle Tracking Analysis

The exosomes obtained through ultra-fast centrifugation were extracted from a 5 uL volume and subsequently diluted to 30 uL before being sent for detection. Following successful completion of the instrument performance test using standard materials, the exosome sample can be applied onto the instrument. It is important to ensure gradient dilution in order to prevent sample blockage within the injection needle. Subsequent analysis using the NanoFCM instrument (flow-bio-flow NanoAnalyzer(Xiamen, China)) will provide information on both particle size and concentration of the detected exosomes.

### 2.5. miRNA Sequencing

The total RNA was extracted using the Trizol reagent (Invitrogen, Carlsbad, CA, USA) according to the manufacturer’s protocol. Subsequently, the quantity and purity of the total RNA were analyzed using a Bioanalyzer 2100 (Agilent, Santa Clara, CA, USA), with a RIN number >7.0. Approximately 1 µg of total RNA was utilized for small RNA library preparation following the TruSeq Small RNA Sample Prep Kits protocol (Illumina, San Diego, CA, USA). Finally, single-end sequencing (36 bp or 50 bp) was performed on an Illumina Hiseq 2500 at LC-BIO (Hangzhou, China), following the recommended protocol provided by the vendor.

### 2.6. Bioinformatics Analysis of miRNA

The raw reads were processed using a proprietary software, ACGT101-miR (LC Sciences, Houston, TX, USA), to remove adapter dimers, irrelevant sequences, low-complexity regions, and common RNA families such as rRNA, tRNA, snRNA, and snoRNA. Additionally, repetitive sequences were also eliminated. Subsequently, unique sequences with a length ranging from 18 to 26 nucleotides were aligned against specific species precursors in miRBase 21.0 through BLAST search for the identification of known miRNAs as well as novel miRNAs derived from both the 3p and 5p arms. During the alignment process, variations in length at both ends of the sequence and one mismatch within the sequence were allowed. Known miRNAs were identified based on unique sequences mapping to specific species of mature miRNAs located in hairpin arms. Novel candidates for 5p or 3p derived miRNAs were determined by identifying unique sequences mapping to the opposite arm of known specific species precursor hairpins that did not contain annotated mature miRNAs. The remaining unmapped sequences underwent BLAST search against other selected species precursors (excluding specific species) in miRBase 21.0, followed by further BLAST analysis against respective genomes to determine their genomic locations if any matches occurred. Through these two above-mentioned approaches, we identified additional potential miRNAs among our data set. For those unmapped sequences that did not yield any significant hits during genome searches but contained flanking regions of approximately 80 nucleotides on either side, we predicted potential hairpin RNA structures using RNAfold software 2.1.9 (http://rna.tbi.univie.ac.at/cgi-bin/RNAfold.cgi (accessed on 27 May 2023)).

### 2.7. Analysis of Differentially Expressed miRNAs

The analysis of miRNAs’ differential expression involved a range of statistical tests such as the Fisher exact test, Chi-squared 2 × 2 test, Chi-squared nXn test, Student’s *t*-test, or ANOVA depending on the experimental design. For each specific test conducted, a significance threshold of either 0.01 or 0.05 was employed.

### 2.8. Prediction of Target Genes of miRNAs

To predict the genes targeted by the most abundant miRNAs, two computational target prediction algorithms (TargetScan 5.0 and Miranda 3.3a) were employed to identify binding sites of miRNAs. Subsequently, the predictions from both algorithms were integrated, and overlaps were calculated. Additionally, the KEGG pathway was annotated for these highly expressed miRNAs’ targets.

### 2.9. Quantitative Real-Time PCR

The exosomal RNAs were isolated from the same serum samples used for the miRNA sequencing. Exosomal RNA was extracted using the exoRNeasy Serum/Plasma Midi Kit (#77044, Qiagen, Hilden, Germany) following the manufacturer’s instructions. Subsequently, cDNA synthesis was performed using the miRcute Plus miRNA First-Strand cDNA Synthesis Kit (#KR211-02, Tiangen Biotech Co., Ltd., Beijing, China), and analysis was conducted using the miRcute Plus miRNA qPCR Detection Kit (SYBR Green) (#FP411-02, Tiangen Biotech Co., Ltd., Beijing, China) as per the manufacturer’s recommendations. For this, 5S RNA served as an internal control in triplicate biological and duplicate technical replicates. Expression levels were analyzed using the 2^−ΔΔCt^ comparative method. The primers for miRNAs and 5S RNA are listed in Table 1; genes no. of miRNAs were obtained from miRBase, while 5S RNA was sourced from GenBank.

### 2.10. Statistical Analysis

The data were presented as the mean ± standard error of the mean (SEM). Differences in the expression of selected genes were assessed using Student’s *t*-test (Excel, Microsoft Corporation, Redmond, WA, USA). Statistical significance was considered at *p* < 0.05, with highly significant at *p* < 0.01.

## 3. Results

### 3.1. Isolation and Characterization of Serum Exosomes

**Figure 1:**
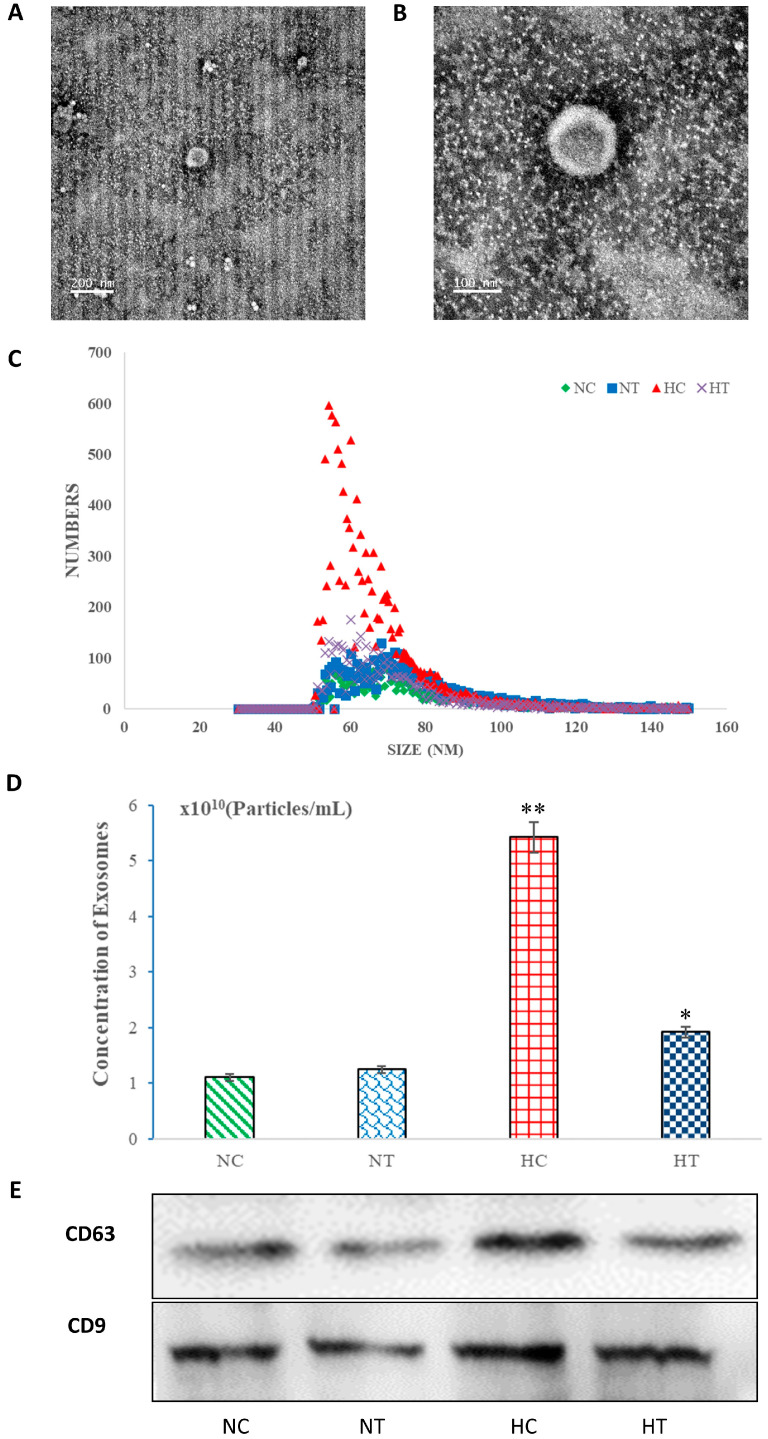
Isolation and characterization of serum-specific exosomes from laying hens in the Control group (NC), Control with curcumin added group (NT), Heat stress group (HC), and Heat stress with curcumin added group (HT) were performed. (**A**,**B**) show electron micrographs displaying whole-mount exosomes isolated from serum, with a scale bar of 200 nm in (**A**) and 100 nm in (**B**). The particle size distribution in exosome-enriched fractions is shown in (**C**). NTA analysis revealed the concentration of serum exosomes, as depicted in (**D**). The data represent three independent experiments and are presented as the mean ± S.E.M (error bars). Statistical significance was determined using two-tailed Student’s *t*-test (* *p* < 0.05; ** *p* < 0.01). WB analysis was conducted to examine the labeled membrane proteins (CD63 and CD9) of exosomes across all four groups (**E**).

The serum exosomes are small, membrane-bound vesicles, as depicted in Figure 1A,B. The observed vesicles exhibited typical exosome size and morphology, with a particle diameter ranging from 30 to 150 nm (Figure 1C). The average diameters of the isolated exosome particles were measured at 76.05, 75.55, 65.98, and 69.88 nm, respectively, while the most widely distributed particle diameters were found to be approximately 68.75, 68.25, 54.25, and 60.25 nm in the NC, NT, HC, and HT groups, respectively (Figure 1C). Furthermore, the average concentrations of exosomes were determined to be at levels of approximately 1.1 × 10^10^ (Particles/mL), 1.24 × 10^10^ (Particles/mL), 5.42 × 10^10^ (Particles/mL), and 1.92 × 10^10^ (Particles/mL) in the NC, NT, HC, and HT groups, respectively. Moreover, the concentration of exosomes was significantly higher in both the HC group (*p* < 0.01) and the HT group (*p* < 0.05) compared to that in the NC group (Figure 1D). Additionally, the presence of the labeled membrane proteins CD63 and CD9 on exosomes was detected across all four groups (Figure 1E).

### 3.2. Differential Expression Analysis of miRNA Profiles in Serum Exosomes

**Figure 2:**
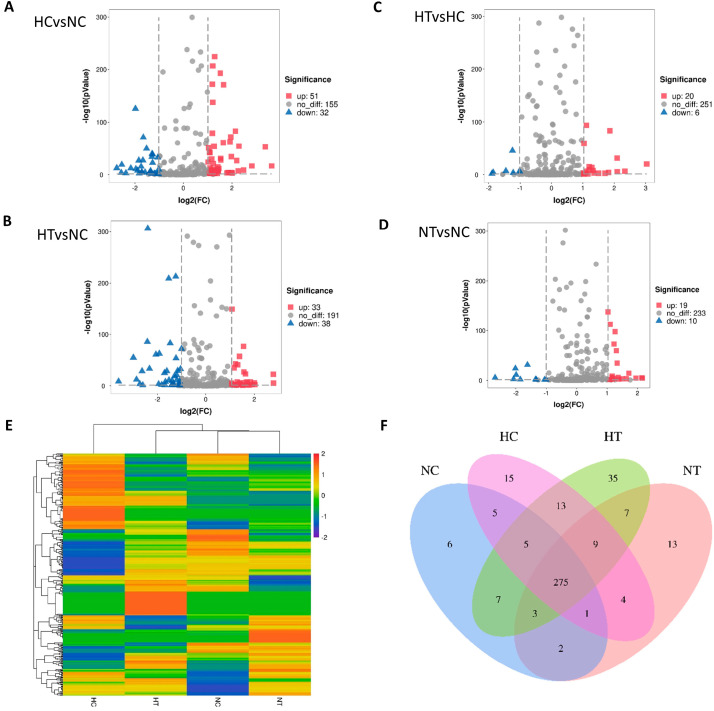
The differential abundance of miRNAs in serum exosomes from laying hens was analyzed in four groups: Control group (NC), Control with curcumin added group (NT), Heat stress group (HC), and Heat stress with curcumin added group (HT). (**A**–**D**): Volcano Plot displaying the differential abundance of miRNAs between HC vs. NC (**A**), HT vs. NC (**B**), HT vs. HC (**C**), and NT vs. NC (**D**) groups. (**E**): Heatmap presenting the differential abundance of miRNAs using RPKM-normalized log2-transformed counts. Up-regulated transcripts are indicated by red color, while down-regulated transcripts are indicated by blue color. (**F**): Venn diagram showing the number of overlapping miRNAs among the four groups.

A total of 863 unique miRNAs were identified from the sRNA libraries, out of which 328 gga-miRNAs (chicken miRNA in miRBase) were specifically identified and utilized for subsequent analysis. The significantly different miRNAs were visualized through a Volcano Plot, where an FC value greater than 2 and a *p*-value less than 0.05 indicated statistical significance (Figure 2A–D). DESeq was employed to assess the enriched miRNA profiles between two groups: the NC, NT, HC, and HT group (Figure 2E). Specifically, there were significant enrichments of 6, 13, 15, and 35 miRNAs in the NC, NT, HC, and HT group, respectively; meanwhile, all groups shared the expression of a common set of 275 miRNAs (Figure 2F).

### 3.3. Bioinformatics Analysis of the Target Genes of the Differentially Expressed miRNAs

The target genes of differentially expressed miRNAs in Figure 2E were predicted and further analyzed in the KEGG database. A comparative analysis of any two of the four groups (NC, NT, HC, and HT) revealed significant differences (*p*-value < 0.01) in four KEGG pathways: cysteine and methionine metabolism, protein export, fatty acid degradation, and oxidative phosphorylation. Interestingly, only the NT vs. NC group exhibited a significant difference (*p*-value < 0.01) in ubiquitin-mediated proteolysis, whereas both ubiquitin-mediated proteolysis and cardiac muscle contraction showed significance (*p*-value < 0.01) only in the HT vs. NC group (Table 2).

### 3.4. RT-qPCR Experimental Validation of the Sequencing Data

**Figure 3:**
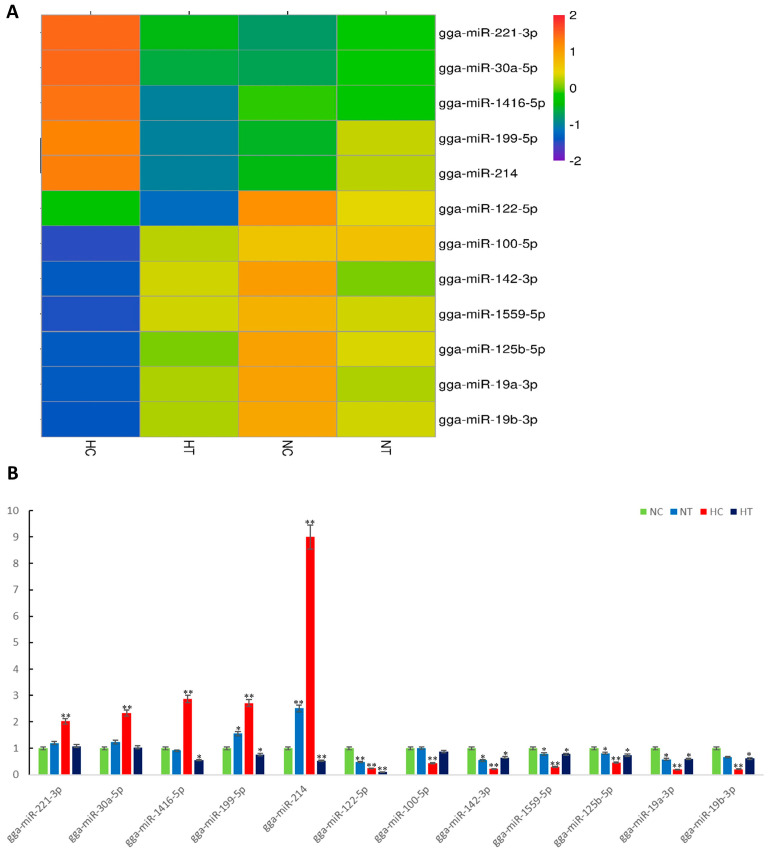
The reliability of the sequencing data by the real-time PCR method. (**A**): Heatmap displaying log2 Fold Change was used to select 12 differentially abundant miRNAs from 4 groups, in order to evaluate the accuracy of the sequencing data using qPCR. (**B**): The qPCR data presented here represent the mean ± S.E.M. of four independent experiments. Statistical analysis was performed using two-tailed Student’s *t*-test, with * indicating *p* < 0.05 and ** indicating *p* < 0.01.

To validate the reliability of the sequencing data, real-time PCR was employed to examine 12 differentially expressed exosomal miRNAs across four groups (Figure 3A). Remarkably, the RT-qPCR analysis confirmed consistent expression patterns for all these miRNAs, as observed in the RNA-seq data (Figure 3B).

## 4. Discussion

In this study, we initially isolated serum exosomes from Roman laying hens that were subjected to heat stress and fed a diet containing either 0 or 200 mg/kg curcumin. Subsequently, we examined the expression profiles of the miRNAs in these exosomes. The numbers of exosomes in the HC and HT groups were significantly higher compared to the NC (control) group. Additionally, the most prevalent particle diameters in the NC, HC, and HT groups were found to be 68.75 nm, 54.25 nm, and 60.25 nm, respectively. This is similar to the other two studies on chicken exosomes: the diameter of exosomes from the bile of 42–43-day-old age-specific pathogen-free chickens can range from 33 nm to 60 nm [2], while chicken type II pneumocytes (CP-II)-derived exosomes have an average particle diameter of 30–150 nm, with a double-layer membrane structure consisting of round vesicles and diameters around 100 nm [7]. Meanwhile, the particle size of exosomes that are purified from the serum of highly pathogenic H5N1 avian influenza virus-infected and -uninfected white Leighang chickens ranged from 105 to 356 nm, with an average size of 141 nm (control) and 145 nm (infected) [8], which were different to the particle size range and average size detected by us. It is speculated that this is related to the breed of experimental chickens and different experimental treatments. Here, the author used highly pathogenic H5N1 avian influenza virus for the challenge treatment [8], and the experimental chickens had a relatively severe stress response. However, exosomes from other animals exhibit different sizes. In cows, the vesicles have an average particle diameter size of 30–140 nm. On average, the isolated exosome particles measure 121 nm or less in diameter, with a widely distributed particle diameter peak at 118.4 nm [1]. In rats, the main peaks for isolated particle sizes are at 66, 138, and 260 nm, respectively; more than 70% of these particles have diameters ranging from 30 to 150 nm [4]. For humans, the diameter distribution of exosomes ranges approximately from 50 to 150 nm in diameter [7]. These findings suggest that serum exosome specifications also vary across different species under various experimental conditions.

Curcumin(1, 7 (4-hydroxy-3-methoxy phenyl)-1, 6-heptyl diene-3, 5-diketone), a chemical compound that is found in turmeric rhizome, possesses the ability to rapidly penetrate lipid molecules within cell membranes. It can suppress signaling molecules that are involved in regulating cell apoptosis and prevent reactive oxygen species (ROS), among other mechanisms that regulate various molecular pathways within cells [20]. Curcumin enhances the expression levels of oxidative stress markers, ovarian reserve marker gene expressions, and histopathological parameters to reduce ovarian damage. It significantly improves the pathological damage that is caused by stress models on ovarian tissue and abnormal hormone levels [21]. Heat stress induces oxidative stress in liver tissues and diminishes immune responses in laying hens, leading to diseases that affect poultry production performance. However, heat-stressed hens who are supplemented with dietary curcumin exhibit enhanced immunity against stressful environmental conditions [22]. 

miRNA regulation of oxidative stress-induced cellular aging is considered a permanent state wherein growing cells are halted under stressful conditions. The free radical theory of aging, also known as the “theory of oxidative stress (OS)”, is currently one of the most extensively studied mechanisms promoting aging. Various epigenetic studies have revealed that microRNAs play a crucial role in controlling OS during cell senescence [23]. The statistical analysis of the experiment conducted on laying hens in the chicken group revealed that the curcumin protection group exhibited a significantly higher rate of high-temperature stress, approaching the level of the room temperature control group. A total of 286 gga-miRNAs (chicken miRNA) were identified and utilized for subsequent analysis. Among them, 8, 19, and 18 miRNAs were found to be significantly enriched in the NC, HC, and HT groups, respectively; meanwhile 1, 10, and 6 miRNAs were enriched in comparisons between NC vs. HC, HC vs. HT, and HT vs. NC, respectively. Notably, there were significant differences observed between HC vs. NC, with an up-regulation/down-regulation ratio of 51:32; similarly, there were also significant differences detected between HT vs. HC, with an up-regulation/down-regulation ratio of 33:38; and finally, there were significant differences detected between HT vs. NC, with an up-regulation/down-regulation ratio of 20:6.These findings suggest that certain crucial miRNAs are involved in regulating the thermal stress response in laying hens, as well as mediating curcumin-induced alleviation of heat stress, during which laying hens adapt to heat stress conditions while maintaining a stable egg production.

The target genes that are expressed by different miRNAs in the three groups were analyzed in the KEGG database. There were four KEGG pathways (oxidative phosphorylation, protein export, cysteine and methionine metabolism, and fatty acid degradation) with a *p*-value < 0.01 observed between each pair of groups. Additionally, two distinct pathways were exclusively identified in specific group comparisons: ubiquitin-mediated proteolysis (both in HT vs. HC and HT vs. NC) and cardiac muscle contraction (only in HT vs. NC). Both heat stress and curcumin can regulate or influence these aforementioned pathways. Heat stress stimulates reactive oxygen species (ROS) production in birds’ mitochondria, where enhanced substrate oxidation plays a crucial role in ROS overproduction for heat-stressed birds, possibly through elevated mitochondrial membrane potential [24]. 

Curcumin exhibits characteristics that are similar to classic uncouplers like 2,4-dinitrophenol; it has been found to modulate mitochondrial respiration, which is a characteristic feature of inhibitory uncouplers [25]. Furthermore, extensive experimental and theoretical investigations have identified CRM1 as an important cellular target of curcumin for nuclear exportation [26]. Heat stress induces changes in the fecal microbiome and functional pathways of laying hens. The functional prediction of these alterations in the microbiota reveals that metabolism-related pathways, such as cysteine and methionine metabolism [27], are affected. Previous research on curcumin’s impact on cancer cells based on cell metabolic profiling has demonstrated its interference with multiple metabolic pathways, including cysteine and methionine metabolism [28]. During heat stress, late pregnant cows activate the Cahill pathway but reduce Cori cycling to prevent an increase in skeletal muscle fatty acid oxidation. In early lactation, metabolic adaptation to heat stress involves increased degradation of long-chain fatty acids in muscle peroxisomes [29]. The presence of curcumin stimulates the formation of short-chain fatty acids such as butyric and propionic acids, which acted as clinical biomarkers for microbiota modulation in patients with hypertension during an in vitro simulation process [30]. Differentially expressed genes (DEGs) in Pacific abalone Haliotis discus hannai between control and heat stress temperatures are enriched in pathways such as ubiquitin-mediated proteolysis and cardiac muscle contraction [31]. Additionally, curcumin significantly increases p27 protein’s half-life and attenuates ubiquitin proteasome activity in 3T3-L1 preadipocytes [32]. Curcumin, a safe and simple natural compound, exhibits excellent anti-stress and antioxidant properties. When used alone or in combination with other feed additives, curcumin significantly enhances the antioxidant capacity of poultry, mitigates stress damage caused by pathogen infection [16], and effectively protects poultry tissues and organs from oxidative stress damage [17]. Our previous studies have also demonstrated that dietary curcumin improves the antioxidant capacity of laying hens under heat stress and alleviates stress-induced damage [11]; however, its regulatory mechanism remains unexplored.

In this study, we observed that curcumin alters the number, particle size, and miRNA profile of serum exosomes in Roman laying hens who are exposed to both room-temperature and high-temperature conditions. Through differential exosome transport miRNA analysis, our findings suggest that curcumin may exert a protective effect on certain pathways that are associated with serum exosomal miRNA regulation in laying hens under heat stress. Investigating the mechanisms underlying these regulatory pathways is an area for further research.

## 5. Conclusions

In conclusion, heat stress induced distinct alterations in the number, size, and distribution of serum exosomes in Roman laying hens. The expression profiles of miRNAs in serum exosomes were found to be more similar between Roman laying hens under control conditions (NC) and those who were fed a diet with 200 mg/kg curcumin under heat stress (HT). Curcumin was observed to mitigate the impact of heat stress on laying hens by modulating the miRNA expression profile of serum exosomes and associated regulatory pathways. Further research is required to elucidate the functional mechanisms of specific miRNAs that are involved in the aforementioned pathways, such as cysteine and methionine metabolism, protein export, fatty acid degradation, oxidative phosphorylation, etc.

## Figures and Tables

**Table 1:** The primers for qPCR.

Genes	Sequences of Primer (5′–3′)	Genes No.
gga-miR-100-5p	AACCCGTAGATCCGAACTTGTG	MIMAT0001178
gga-miR-122-5p	TGGAGTGTGACAATGGTGTTTGT	MIMAT0001190
gga-miR-125b-5p	TCCCTGAGACCCTAACTTGTGA	MIMAT0001105
gga-miR-1416-5p	TCCTTAACTCATGCCGCTGTG	MIMAT0007285
gga-miR-142-3p	TGTAGTGTTTCCTACTTTATGG	MIMAT0001194
gga-miR-1559-5p	TTCGATGCTTGTATGCTACTCC	MIMAT0007416
gga-miR-199-5p	CCCAGTGTTCAGACTACCTGTTC	MIMAT0001152
gga-miR-19a-3p	TGTGCAAATCTATGCAAAACTGA	MIMAT0001112
gga-miR-19b-3p	TGTGCAAATCCATGCAAAACTGA	MIMAT0001110
gga-miR-214	ACAGCAGGCACAGACAGGCAG	MIMAT0007751
gga-miR-221-3p	AGCTACATTGTCTGCTGGGTTTC	MIMAT0001108
gga-miR-30a-5p	TGTAAACATCCTCGACTGGAAG	MIMAT0001135
gga-5s-rRNA-F	CCATACCACCCTGGAAACGC	AF419700
gga-5s-rRNA-R	TACTAACCGAGCCCGACCCT	AF419700

**Table 2:** The KEGG pathways of the target genes of differentially expressed miRNAs.

Groups	Pathway Id	Pathway Description	S Gene Number	TS Gene Number	*p* Value of Fisher’s Exact Test
NT vs. NC	ko00270	Cysteine and methionine metabolism	27	1900	1.15 × 10^−3^
	ko03060	Protein export	16	1900	1.17 × 10^−3^
	ko00071	Fatty acid degradation	22	1900	1.41 × 10^−3^
	ko00190	Oxidative phosphorylation	57	1900	1.66 × 10^−3^
	ko04120	Ubiquitin-mediated proteolysis	63	1900	7.03 × 10^−3^
HC vs. NC	ko00190	Oxidative phosphorylation	60	1925	3.07 × 10^−4^
	ko03060	Protein export	16	1925	1.38 × 10^−3^
	ko00270	Cysteine and methionine metabolism	27	1925	1.46 × 10^−3^
	ko00071	Fatty acid degradation	22	1925	1.74 × 10^−3^
HT vs. NC	ko00071	Fatty acid degradation	23	1877	3.27 × 10^−4^
	ko00190	Oxidative phosphorylation	58	1877	5.93 × 10^−4^
	ko00270	Cysteine and methionine metabolism	27	1877	9.22 × 10^−4^
	ko03060	Protein export	16	1877	9.95 × 10^−4^
	ko04120	Ubiquitin-mediated proteolysis	63	1877	5.09 × 10^−3^
	ko04260	Cardiac muscle contraction	30	1877	9.22 × 10^−3^
HT vs. HC	ko00071	Fatty acid degradation	23	1897	3.93 × 10^−4^
	ko00190	Oxidative phosphorylation	59	1897	4.01 × 10^−4^
	ko00270	Cysteine and methionine metabolism	26	1897	3.08 × 10^−3^
	ko03060	Protein export	15	1897	4.69 × 10^−3^

S gene number: the number of genes that are significantly different for a particular KEGG; TS gene number: the number of genes that are significantly different.

## Data Availability

All relevant data are included within the paper.
